# The Importance of Social Determinants of Health for Cancer Patients in the Era of COVID-19

**DOI:** 10.7759/cureus.27993

**Published:** 2022-08-14

**Authors:** Barat S Venkataramany, Jeffrey M Sutton

**Affiliations:** 1 Department of Surgery, The University of Toledo College of Medicine and Life Sciences, Toledo, USA; 2 Division of Oncologic and Endocrine Surgery, Department of Surgery, Medical University of South Carolina, Charleston, USA

**Keywords:** covid-19 retro, health care delivery, health care disparity, epidemiology and public health, social determinants of health (sdoh)

## Abstract

The COVID-19 pandemic has proven to be a challenge for public health professionals, researchers, clinicians, and patients. One group that has experienced significant difficulties during this time is cancer patients. Data regarding this vulnerable population is scarce, despite novel information about vaccine efficacy, therapeutics, mutations, and comorbidities. In this article, we discuss the need for a greater study of social determinants of health (SDOH) for cancer patients in the context of the COVID-19 pandemic. The effects of SDOH on population health are generally well-understood, but their effects on cancer patients are poorly understood. We further pose questions that may be starting points for the investigation of SDOH in cancer patients during this time. Using SDOH as a tool for more effective clinical care will promote the development of targeted interventions to study and improve outcomes in this population.

## Editorial

From exposing the shortcomings of medical infrastructure to challenging the efficacy of public health messaging, the COVID-19 pandemic has unquestionably transformed the landscape of healthcare in a multitude of ways. Each group of patients and professionals alike have experienced numerous difficulties over the course of the pandemic. Cancer patients represent a vulnerable population that has faced a distinct set of challenges in this time. Like other strata of patients with underlying pre-existing medical conditions, this unique group is especially susceptible to infection and poorer outcomes as a result of COVID-19 [1]. However, the limited body of data on this subset of patients precludes us from understanding exactly why this group is predisposed to poorer outcomes. For this reason, we must assess the role of social determinants of health (SDOH) in cancer-related outcomes.

While the effects of SDOH on population health are generally well-characterized, their effects on cancer patients are considerably less studied. This paradigm takes on greater importance in the era of COVID-19; certain populations, such as those with lower socioeconomic status (SES) or with underlying comorbidities, have been shown to be much more vulnerable than others, though the reasons remain poorly understood [1]. Associations between cancer outcomes and both SES and chronic comorbidities (e.g., cardiovascular disease) have been well-documented [2,3] and cannot be discounted when assessing outcomes for cancer patients during this time. Such relationships may be products of a complex interplay between societal and biological factors that contribute to the development of chronic disease. SDOH variables may trigger adverse health outcomes due to their ability to act synergistically (e.g., SES may be determined by education and employment opportunities that arise as a result of educational level), but determining the degree to which these factors interact, both with each other and with factors intrinsic to patients (e.g., genetics), is difficult due to the interrelatedness of variables.

The notion that assessing such relationships requires further study brings us to a recurring theme concerning cancer patients and COVID-19: the need for more data. As Disis noted, we may try to explain the phenomena as the greater risk of infection in cancer patients with theoretical reasons (e.g., a pre-existing immunosuppressed state that prevents the generation of an adequate immune response), but clinical data to support these hypotheses are lacking [2]. Without the clinical knowledge upon which we predicate our practice of evidence-based medicine, we cannot hope to deliver the best possible care for an especially vulnerable population. Just over two years after the onset of the pandemic, clinicians and consortia are still establishing the best practices for caring for cancer patients [4,5]. And when attempting to treat an unpredictable disease like cancer, each weapon in the screening and treatment arsenal is crucial; not considering SDOH as a factor when designing treatments unique to each patient will represent a major failure on the part of physicians and health systems. It is therefore imperative that we integrate the study of SDOH into such decision-making processes so that we can continue to mitigate the risk of adverse outcomes in this population.

The first results reported by the CCC19 group represented an encouraging development in the investigation of COVID-19-related outcomes for cancer patients through the large-scale study of variables ranging from age and smoking status to type of malignancy and treatment received for COVID-19. At the time of publication, this was the largest series that analyzed the outcomes for cancer patients with COVID-19 (928 patients) and found a 30-day all-cause mortality rate of 13% [6]. As the study was one of the first to investigate the risk factors for cancer patients during the pandemic, the authors noted a need for more data as well as further research into how factors such as occupational exposure and healthcare provider availability, among others, affect mortality from COVID-19 [6]. The consortium remains active in its study of COVID-19 in cancer patients and continues to assess how a variety of factors influence outcomes in this population. But broader categories of SDOH, such as economic stability and access to health care, require a much more detailed examination in the context of the pandemic for this population. The facts that we know about the effects of different SDOHs on cancer-related outcomes will be integral as we attempt to understand the outcomes for cancer patients during this time. For example, SES, housing instability, and discrimination have all previously been associated with poor access to cancer screening [3]. But the paucity of data concerning such SDOH and cancer-related outcomes in the pandemic raises the question: What is the link between a cancer patient’s circumstances during the COVID-19 pandemic and their risk of mortality from cancer? We may examine certain variables through a societal lens.

Questions for further investigation

A series of examples and resultant questions that may be posed to further study the effects of selected SDOH on cancer patients in the pandemic are discussed in the following.

The en masse postponement/cancellation of procedures, unforeseen changes to treatment regimens, and sudden migration to telemedicine platforms significantly disrupted care across the world [4]. While such constraints are the results of decisions taken to attenuate the burden placed on healthcare systems amidst a global state of emergency, the effects of these decisions on cancer-related outcomes cannot be overstated. How did these decisions affect the receipt/utilization of different kinds of care? And what underlying characteristics of patients may be associated with any observed differences in access to care?

The economic consequences of the COVID-19 pandemic include concerns regarding food insecurity, unemployment, and housing instability. Due to the association between economic stability and SES as well as the relationship between SES and cancer-related outcomes [3], cancer patients may be especially susceptible to poorer outcomes during this time. Which economic characteristics of patients are associated with the differences in access to cancer care and outcomes during the COVID-19 pandemic?

Misinformation in social and news media has contributed to eroding levels of trust in scientific, public health, and governmental institutions [7]. As a result, patients with decreased levels of literacy (both general and health literacy) may experience difficulties in discriminating between fact-based findings and fake news related to the COVID-19 pandemic. What are the associations between the levels of literacy in these situations and cancer-related outcomes?

Disparities in accessing preventative care (i.e., cancer screening) are well-characterized and significant [3]. We must consider that these differences may have been considerably exacerbated by the need to stay at home and delay care. What characteristics of patients are associated with differential access to cancer screening during the COVID-19 pandemic? How do these demographics affect risk and mortality?

Recently, Taquet et al. found in a study of over 200,000 COVID-19 survivors that 23.98% of patients received a diagnosis of any mood, anxiety, or psychotic disorder within six months of COVID-19 infection [8]. Given that higher rates of depressive symptoms are associated with greater rates of mortality from cancer [9], how do the findings of this study translate to cancer patients in particular? What are the underlying characteristics that may predispose cancer patients to poorer mental health outcomes during this time? And how does a patient’s level of social support during a time of increased isolation correlate with cancer-related outcomes?

We recognize that neither of these are the only questions to be posed nor are the only variables that should be investigated. Additionally, SDOHs are myriad in nature and often interact with one another to produce a particular outcome, which may complicate the selection of factors to be studied in the greatest depth. Recently, Abrams and Szefler noted how poverty, living environment, environmental exposure, and food insecurity may together induce a substantial adverse effect on COVID-19-related outcomes [10]. This is but a single example that can be studied in the context of the pandemic. But the study of SDOH is not a silver bullet by which we might immediately answer the majority of questions surrounding cancer patients and COVID-19. As each day brings new findings on virulence and vaccine efficacy that help to drive international public health messaging and governmental decisions, research into SDOH and outcomes for cancer patients can have similar effects and improve the ability of clinicians to provide the best possible care. We must probe further to understand why certain strata of patients experience adverse outcomes at disproportionate rates compared to others. Like many of the effects of COVID-19, the effects of the pandemic on cancer patients are either poorly understood or are in the infancy of being elucidated, but answering questions surrounding SDOH and this patient population will enable the development of targeted interventions to alleviate disparities and improve both care and outcomes. As we increasingly study how this global health crisis has affected different groups of patients, perhaps an individual’s experience during the COVID-19 pandemic will serve as an SDOH in its own right and prove important for successful health outcomes.

## References

[1] Wang Q, Berger NA, Xu R (2021). Analyses of risk, racial disparity, and outcomes among us patients with cancer and COVID-19 infection. JAMA Oncol.

[2] Disis ML (2020). Oncology and COVID-19. JAMA.

[3] Alcaraz KI, Wiedt TL, Daniels EC, Yabroff KR, Guerra CE, Wender RC (2020). Understanding and addressing social determinants to advance cancer health equity in the United States: a blueprint for practice, research, and policy. CA Cancer J Clin.

[4] van de Haar J, Hoes LR, Coles CE (2020). Caring for patients with cancer in the COVID-19 era. Nat Med.

[5] Bartlett DL, Howe JR, Chang G (2020). Management of cancer surgery cases during the COVID-19 pandemic: considerations. Ann Surg Oncol.

[6] Kuderer NM, Choueiri TK, Shah DP (2020). Clinical impact of COVID-19 on patients with cancer (CCC19): a cohort study. Lancet.

[7] Narayan KM, Curran JW, Foege WH (2021). The COVID-19 pandemic as an opportunity to ensure a more successful future for science and public health. JAMA.

[8] Taquet M, Geddes JR, Husain M, Luciano S, Harrison PJ (2021). 6-month neurological and psychiatric outcomes in 236 379 survivors of COVID-19: a retrospective cohort study using electronic health records. Lancet Psychiatry.

[9] Pinquart M, Duberstein PR (2010). Depression and cancer mortality: a meta-analysis. Psychol Med.

[10] Abrams EM, Szefler SJ (2020). COVID-19 and the impact of social determinants of health. Lancet Respir Med.

